# Risk of Covid-19 infection during endoscopy: Efficacy of personal protective equipment (PPE) in protecting health professionals

**DOI:** 10.12669/pjms.37.4.4057

**Published:** 2021

**Authors:** Muhammad Akif Dilshad, Shahid Sarwar, Farheen Aslam, Adnan Shabbir, Shafqat Rasool, Ghias un-Nabi Tayyab

**Affiliations:** 1 Dr. Akif Dilshad, MBBS, FCPS (Gastroenterology). Associate Professor Gastroenterology, Lahore General Hospital, Lahore, Pakistan; 2 Dr. Shahid Sarwar, MBBS, FCPS (Medicine), FCPS (Gastroenterology), MCPS-HPE, FRCP (Edin) Professor of Medicine, Allama Iqbal Medical College, Lahore, Pakistan; 3 Farheen Aslam, PhD. Assistant Professor, Department of Biotechnology, Lahore College for Women University, Lahore, Pakistan; 4 Adnan Shabbir, MBBS. Post Graduate Resident Gastroenterology Lahore General Hospital, Lahore, Pakistan; 5 Shafqat Rasool, MBBS, FCPS (Gastroenterology). Assistant Professor Gastroenterology Lahore General Hospital, Lahore, Pakistan; 6 Ghias Un Nabi Tayyab, MBBS, MRCP (UK) FCPS, FRCP (Edin) AGAF (USA). Professor of Medicine, Lahore General Hospital, Lahore, Pakistan

**Keywords:** Covid-19 infection, Endoscopy, Personal Protective equipment (PPE)

## Abstract

**Objectives:**

To correlate compliance to personal protective equipment (PPE) protocols and risk of exposure to SARS-COV-2 infection in endoscopy staff.

**Methods:**

We included 85 endoscopic procedures performed at Lahore General Hospital from May to July 2020. Standard operating procedures (SOPs) were implemented for patient selection, risk stratification and personal protective equipment (PPE) use for endoscopy staff. Patient and endoscopy staff were followed for Covid-19 infection. PPE scores for staff and Covid-19 positivity on follow-up were correlated using student’s t test.

**Results:**

Following 85 procedures included, 2 (2.3%) patients became Covid-19 positive. PPE score was <9 in 5 (5.8%) procedures for endoscopist and Covid-19 developed in 2 (2.3%) of them, PPE score was <9 during 19 (22.3%) procedures in 1^st^ assistant and 9 (10.5%) developed infection and for 2^nd^ assistant PPE score was <9 in 19(22.3%) endoscopies and 5 (5.9%) tested positive for covid-19. Infectivity of endoscopy staff was 6.2%. Association between PPE score and risk of Covid-19 was not significant. (p value 0.51 for endoscopist, 0.10 for 1^st^ assistant and 0.09 for 2^nd^ assistant).

**Conclusion:**

Compliance of SOPs for infection control reduces risk of acquiring Covid-19 infection during endoscopy. Proper use of PPE is effective for safety of endoscopy staff.

## INTRODUCTION

Severe acute respiratory tract syndrome following exposure to corona virus 2 (SARS COV-2) is responsible for viral pneumonia labeled as COVID-19 infection. It was first discovered in last month of 2019^1^, however due to highly infectious potential and presence of asymptomatic patients, it quickly transformed in to a pandemic.^2^ People in more than 212 countries have reported exposure to this infection and confirmed reported cases are more than 65 million with more than 1.4 million deaths.^3^

Covid-19 infection spreads either by direct contact with or inhalation of respiratory air droplets. Infected person can spread this disease either by touching infected hands on different surfaces from where healthy individuals are exposed to this virus by touching same surfaces and then touching their nose, mouth or eyes with infected hands or by shedding these viruses in air while sneezing or coughing. 4 These viruses in air droplets or aerosols can remain suspended in air for hours and may enter in healthy person with breathing, if in close proximity to infected individual.^5^

Due to exponential spread of this highly infectious and lethal viral infection, routine clinical services including diagnostic and therapeutic procedures were immediately curtailed.^6^ Many clinical procedures like nebulization, endotracheal intubation, bronchoscopy, open suctioning, ventilatory support, noninvasive positive-pressure ventilation, tracheostomy and endoscopy are potentially aerosol generating procedures and can results in spread of this infection.^7^

In addition to risk of transmission through infected aerosols being generated during procedure, in endoscopy room COVID-19 can also spread via direct person to person contact or while handling contaminated endoscopic equipment, accessories and body fluids.^8^ Endoscopy suits are generally small, closed units where multiple persons including endoscopists, nursing staff, technician, anesthetist and hospital attendants are present. Patients with their attendants may stay in waiting rooms for many hours. Even a single patient of Covid-19 infection in this setting can contaminate suits air with viruses which may remain suspended in air for many hours.^9^ Moreover, fecal shedding of virus in a patient further adds to the risk during colonoscopy.^10^

Despite viral infection still rampant world-over even after more than nine months of being declared as pandemic by WHO, diagnostic and therapeutic endoscopic services have gradually resumed as was inevitable. In order to ensure safety of endoscopic staff during endoscopic procedures, GI societies have developed guidelines regarding patient screening, use of personal protective equipment (PPE) during procedures along with safety standards for handling of equipment and accessories.^11^ Endoscopy staff is required to use face mask, gown, face or eye shield and head and foot covers during procedures with due care for following standard operating procedures (SOP) during donning and doffing of PPE.^6^

However, efficacy of these measures in protecting endoscopy staff from COVID-19 infection still needs to be gauged and will only be known over time. We therefore planned an observational study to correlate compliance to PPE protocols and risk of SARS-COV-2 infection in endoscopy staff.

## METHODS

We planned an observational study at Endoscopy suit of Lahore General Hospital from May 2020 till July 2020, peak time of pandemic in Pakistan when only emergency endoscopic services were available at few tertiary care centers. Our center decided to start limited number of urgent procedures after developing Standard Operating Procedures (SOPs) for selection of patient for procedure, number of personals allowed in procedure room, PPE for endoscopist and procedure assistants. These SOPs were based on recommendation of American Society of Gastrointestinal Endoscopy (ASGE).^11^

Every patient enrolled for endoscopy was stratified as High risk, Intermediate risk and low risk for Covid-19 infection based on

- Fever (Temperature > 37.5 C) in last 14 days
- Exposure to family member or close contact with suspected or confirmed Covid-19 infection
- Belongs to or has travelled to area with higher risk for Covid-19 infection in last 14 days

Patients with none of these features were low risk, patients with fever alone or those with no fever but both exposures to suspected or confirmed patient and resident of high risk area were intermediate risk patients while patient with fever and one of other two features were high risk patients.

For low-risk patient, endoscopy staff were advised to wear hairnet, goggles, surgical mask, single use gown and one pair of gloves while for intermediate and high-risk patients, PPE used included hairnet, goggles/face shield, N95 or Filtering face piece (FFP) II or III face mask with two pairs of gloves. Endoscopy staff was required to wash their hands with soap and water or alcohol-based rub before and after each contact with patient, contact with infectious material and during donning and doffing of PPE. Endoscopy staff was advised to keep reasonable distance from patient while taking consent, checking vitals and giving procedure related instructions.

Temperature of each patient was checked before entering endoscopy room. No relative or caregiver was allowed in suit unless essential for specific assistance. All patients were offered surgical mask while intermediate or high-risk patients also wore gloves before entering endoscopy suit. Surgical mask was removed just before starting procedure once patient was under conscious or deep sedation. It was replaced on face once patient had recovered from sedation and had oxygen saturation >90%.

Compliance of these SOPs was ensured by filling of proforma in real time during procedure that included information regarding indication for procedure, details of patient’s risk stratification, duration of procedure and time of patient stay in endoscopy suit and PPE used by each person present in suit. Patients undergoing procedure and endoscopy staff involved in procedure were followed up on phone at day-7 and day 14 after procedure for development of symptoms suggestive of Covid-19. Patients were advised Covid-19 testing after two weeks while endoscopy staff with suspected symptoms were tested for COVID-19 infection and were provided care as per standard protocols.

### Statistical analysis

Data was analyzed using SPSS 22^®^ (Armonk NY:IBM corp). Quantitative variables were expressed as mean ± standard deviation (SD) whereas qualitative variables were given as percentage.

Personal protective equipment (PPE) used by endoscopist, 1^st^ assistant and 2^nd^ assistant were scored giving two marks each for mask, gloves and goggles/face shield, 2 marks for Tyvek kit while one score for disposable gown, one score each for hairnet and shoe cover. Maximum score possible was 10. Scores were deducted, two each for not wearing mask and gloves, one each for absence of face shield/goggles and gown. PPE score of six or less was considered inadequate score. PPE scores of endoscopy staff, duration of procedure and stay time of patient in suit were correlated with Covid-19 infection in staff during 14 days of follow up using unpaired student’s t test while type of procedure and risk category of patient were analyzed for association with Covid-19 infection using chi square x^2^ test. Mann Whitney U test was used for non-parametric variables.

## RESULTS

We included total of 85 procedures carried out at Lahore General Hospital during three months. It included 53 (62.4%) upper GI endoscopies, 13 (15.3%) colonoscopies and 19 (22.4%) Endoscopic Retrograde Cholangiopancreaticography (ERCP). Procedures performed were of acute emergency in 28 (32.9%) patients, urgent in nature in 51(60%) patients and elective only in six (7.1%) cases. Major indications for procedures included upper GI bleeding in 28(32.9%) patients, obstructive jaundice in 17 (20%) patients, dysphagia in 13 (15.3%) patients, bleeding per rectum in six (7.1%) patients and abdominal pain in five (5.9%) patients. Mean age of patients was 44.89 (±15.6%) and it included 52(61.2%) male patients and 33 (38.8%) females.

On pre-procedure screening, eight (9.4%) patients had history of fever, six (7.1%) complained of cough and three (3.5%) had travel history to high-risk area as well. Total of 10 (11.8%) procedures were of intermediate risk category while rest were low risk. However, we tested 61 (71.8%) patients for Covid-19, out of which only two were positive who were treated as high-risk patients with level one protective measures.

Deep sedation with propofol was used for 81 (95.4%) patients while four (4.8%) underwent procedure with conscious sedation. Endoscopic variceal band ligation was done in 21 (24.7%) patients, six (7.1%) had stricture dilatation, biliary stenting was performed in 17 (20%) patients, biliary stones were removed in two (2.4%) patients and diagnostic biopsy was taken in eight (9.4%) patients. Mean duration of procedures was 24.4 (±17.8) minutes while mean patient stay time in endoscopy suit was 34.64 (±24.6) minutes.

Total number of persons in endoscopy room during procedure apart from patient himself were three in 18 (21.2%) procedures, four in 55 (64.7%) and five in 12 (14.1%) procedures. Mean PPE score for endoscopists in 85 procedures was 9.74 (±0.95), for 1^st^ assistant PPE score was 8.95 (±1.9) and 8.59 (±2.7) was mean PPE score for 2^nd^ assistant.

On two weeks follow up, Covid-19 test was carried out in 73 (85.9%) patients and only two were positive. Both patients recovered without hospitalization and none reported symptoms or positive test in family members. Covid-19 testing was done in endoscopists of 19 procedures due to minor symptoms and two (2.3%) were positive, while endoscopists of remaining procedures were asymptomatic. Both positive cases recovered without need for hospital admission. Similarly out of 85 procedures, for 1^st^ assistant of 27 (31.8%) procedures Covid-19 test was performed and nine (10.5%) were positive. Fortunately, all nine members recovered without need for medical treatment. Testing was needed only for nine (10.5%) 2^nd^ assistant members due to symptoms and five (5.9%) turned out to be positive with eventless recovery from Covid-19 infection.

These 16 Covid-19 infection events were documented in 12 procedures. We compared procedure related variables between procedures resulting in Covid-19 infection in any staff member with procedures where no one got infected as shown in Table-I.

**Table-I:** Comparison of procedures resulting in Covid-19 infection in endoscopy staff with procedures with no staff infection

Variable	Procedures resulting in COVID-19 infection in staff (n-12) Mean± SD	Procedures with no COVID-19 infection in staff (n-73) Mean± SD	P value
Patient age (Years)	43.7 (±14.7)	44.35 (±11.08)	0.81
Procedure time (min)	21.9 (±15.10)	37.9 (±27.1)	0.72*
Patient stay in endoscopy suit (minutes)	32.9 (±22.3)	57.0 (±34.54)	0.39*
No of persons in endoscopy suit	3.75 (±0.86)	4.1 (±0.55)	0.17
Endoscopist PPE score	9.0 (±1.8)	9.5 (±1.23)	0.36
Assistant 1 PPE score	6.58 (±2.8)	7.85 (±2.2)	0.17
Assistant 2 PPE score	6.75 (±3.7)	6.20 (±3.47)	0.67

* : Mann Whitney U test

Mean PPE score in Covid-19 positive endoscopists was 10 as compared to 9.2(±1.65) in those with no infection (p value 0.51). For 1^st^ Assistants mean PPE score for positive persons was 6.22 (±2.53) as compared to 7.94 (±2.4) in those without Covid-19 infection. (p value 0.10). Mean PPE score for Covid-19 positive 2^nd^ assistants was 6.2 (±3.84) as compared to full 10 for those free of infection. (p value 0.09).

On individual evaluation of nine 1^st^ assistants with Covid-19 infection, two had PPE score of 3, one had 5 score, three had 6 score, one had 7 score and two had full 10 score. PPE score of 6 or less was significantly associated with risk of infection in 1^st^ assistant. (p value 0.000) Similarly of 5 infected 2^nd^ Assistants, two had 2 score and three had 5 score and correlation of ≤ 6 score with Covid-19 infection was not significant in 2^nd^ assistant. (p value 0.10) However both infected endoscopists had full 10 PPE score. Therefore, despite variable correlation between PPE scores and Covid-19 infection, majority of staff members who developed Covid-19 infection had sub-optimal personal protective equipment during procedure.

## DISCUSSION

Despite being rampant in almost every country of the world for more than one year with health services and economy of even developed countries being paralyzed, only effective remedy for Covid-19 infection is prevention. Social distancing, frequent hand washing and isolation of infected individuals are THE effective tools for controlling its spread in community.^12^ Health care workers, due to their regular close contact with patients especially during interventional procedures like surgeries and endoscopies have very high risk of catching infection.^13^ PPE and strict infection control measures are therefore essential during procedures in healthcare setting to mitigate this risk.^14^

We, in this observational study have evaluated compliance of standard operating procedures (SOPs) developed in light of ASGE guidelines for endoscopic procedures. However, despite triage protocols and patient screening, endoscopy staff can still be exposed to Covid-19 infection as significant number of Covid-19 patients are asymptomatic. PPE use is warranted to control this risk. We have also determined the efficacy of PPE in saving endoscopy staff from getting infection.

Covid-19 infection transmission was noted in 12 (14.1%) of total 85 procedures carried out in time of peak infection in region. Despite statistically non-significant association between PPE score and Covid-19 infectivity in endoscopy staff, Covid-19 infection rate was 2.4% (2/85) in endoscopists, for whom PPE score less than 9 was observed in only 5.8% (5/85) procedures and infection rate increases to 10.6% (9/85) in 1^st^ assistant and 5.9% (5/85) in 2^nd^ assistant where PPE<9 was seen in 22.3% (19/85) procedures for both 1^st^ and 2^nd^ assistants.

Elective endoscopic procedures were cancelled at start of pandemic due to risk of infection and shortage of PPE in line with international consensus statements.^15^ However it can’t continue indefinitely as a modelling study from UK has estimated that Covid-19 pandemic may result in rise in cases of colorectal cancer and esophageal cancer by 16% and 6% respectively in coming five years.^16^ It is estimated that social distancing to avoid Covid-19 infection may need to be kept in practice till 2022.^17^ Therefore we need to resume these life-saving services with due precautions

A survey from Italy reported only 1 out of 802 patients undergoing endoscopy to be Covid-19 positive on 2 weeks post-procedure follow up. They reported only 4.3% Covid-19 infections among endoscopy staff during these procedures.^18^ We have noted 6.2% infection rate among endoscopy staff for 85 procedures at our center. Similarly, the SCOTS project has reported no Covid-19 infection in patients after two weeks of endoscopy with COVID-minimized pathway implementation.^19^

In a study of more than 99,000 health care workers in UK and USA, it was estimated that health professionals have more than 10-fold risk of catching COVID-19 infection (HR 11.61, 95% CI 10.93-12.33) as compared to individuals in community. It was further concluded that adequacy of PPE can significantly curtail this risk.^20^ However, effective implementation of these protective measures is needed to ensure safety of health professionals who as a community are struggling hard to continue quality healthcare delivery even during pandemic. 21

### Limitations of the Study

We followed patients and endoscopy staff for symptoms of Covid-19 infection for 2 weeks post-procedure. As disease is also prevalent in community, study population can be exposed to virus outside endoscopy suit as well. Therefore, it is impossible to be certain that those infected, were exposed during endoscopy. We tested almost all patients undergoing procedure for Covid-19 infection 2 weeks after procedure but for endoscopy staff, PCR testing for infection was only performed for symptomatic individuals and it risks under-reporting of disease as a significant percentage of infected cases remain asymptomatic. Testing each staff member after procedure was not possible due to limited supply of testing kits in this pandemic and that too for screening. Our study lacks control group with no PPE during procedures, however it is not practical for a highly infectious and potentially lethal disease like Covid-19.

Despite these limitations, risk of Covid-19 infections in endoscopy staff can be avoided by effective implementation of safety protocols and appropriate PPE use by endoscopy staff needs to be assured.

## CONCLUSION

Compliance of standard operating procedures for infection control reduces risk of acquiring COVID-19 infection during endoscopy. Proper use of PPE is effective for safety of endoscopy staff

### Author’s contribution:

**AD:** Conception and design, data acquisition, approval of the version, agreement to be accountable for all aspect.

**SS:** Conception and design, analysis and interpretation, drafting of article, approval of the version, agreement to be accountable for all aspect.

**FA:** Conception, data analysis, revising manuscript critically, final approval of the version and agreement to be accountable.

**AS:** Designing of study, data acquisition, revising manuscript critically, final approval of the version and agreement to be accountable.

**SR:** Conception of study, data acquisition, revising manuscript critically and agreement to be accountable.

**GNT:** Conception and design of study, revising manuscript critically, final approval of the version and agreement to be accountable.
